# Assessment of Primitive Reflexes in High-risk Newborns

**DOI:** 10.4021/jocmr706w

**Published:** 2011-11-10

**Authors:** Min Sohn, Youngmee Ahn, Sangmi Lee

**Affiliations:** a Department of Nursing, Inha University, Incheon, South Korea

## Abstract

**Background:**

Assessment of primitive reflexes is one of the earliest, simplest, and most frequently used assessment tools among health care providers for newborns and young infants. However, very few data exist for high-risk infants in this topic. Among the various primitive reflexes, this study was undertaken particularly to describe the sucking, Babinski and Moro reflexes in high-risk newborns and to explore their relationships with clinical variables.

**Methods:**

This study is a cross-sectional descriptive study. Sixty seven high-risk newborns including full-term infants required intensive care as well as premature infants were recruited in a neonatal intensive care unit using convenient sampling method. The sucking, Babinski and Moro reflexes were assessed and classified by normal, abnormal and absence. To explore their relationships with clinical variables, birth-related variables, brain sonogram results, and behavioral state (the Anderson Behavioral State Scale, ABSS) and mental status (the Infant Coma Scale, ICS) were assessed.

**Results:**

The sucking reflex presented a normal response most frequently (63.5%), followed by Babinski reflex (58.7%) and Moro reflex (42.9%). Newborns who presented normal sucking and Babinski reflex responses were more likely to have older gestational age, heavier birth and current weight, higher Apgar scores, shorter length of hospitalization, better respiratory conditions, and better mental status assessed by ICS, but not with Moro reflex.

**Conclusions:**

High risk newborns presented more frequent abnormal and absence responses of primitive reflex and the proportions of the responses varied by reflex. Further researches are necessary in exploring diverse aspects of primitive reflexes and revealing their clinical implication in the high-risk newborns that are unique and different to normal healthy newborns.

**Keywords:**

Primitive reflex; High risk infants; Korean; Moro reflex; Sucking reflex; Babinski reflex; The Anderson Behavioral State Scale; Infant Coma Scale

## Introduction

The advances in medical technology and improved neonatal care have profoundly increased the survival of high-risk newborns including full-term infants required intensive care as well as premature infants. Clinicians are encountering smaller and sicker newborns with extremely low birth weight (ELBW) and serious health conditions in the neonatal intensive care unit (NICU). Considering that high-risk newborns experience higher mortality and greater risks of various health and developmental problems, early health assessment for their first course of life is critical. Multidisciplinary-based critical care in the NICU requires a variety of assessment tools to evaluate the conditions of these fragile patients and communicate their condition with families and other health care professionals.

Primitive reflexes are brainstem-mediated, automatic movements which may begin as early gestation week 25-26, and which are fully present at birth in term newborns [1,2]. Assessment of these reflexes is one of the earliest, simplest, and most frequently used assessment tools among health care providers for newborns and young infants [2]. Primitive reflexes start to disappear when the central nervous system matures and voluntary motor activities replace them [2]. The normal age-appropriate response is related to the development of normal motor function of newborns or infants. In general clinical settings, the responses of primitive reflexes have been categorized mostly as dichotomous responses such as normal/abnormal or present/absent. However, primitive reflexes often present various degrees of response, which may be meaningful in the neurological capability to stimulus. Persistent, vigorous, weak, or unsymmetrical responses are closely-linked with neurological impairment in full term [3] and high-risk newborns [2]. Very limited data is available concerning the assessment of primitive reflexes of high-risk newborns in both aspects of PTB or pathologic conditions. While not all primitive reflexes are uniformly present in preterm newborns [2], how the diversity of responses of reflexes are related to clinical conditions and how clinicians can interpret them in a neurological context are unclear. Of the primitive reflexes, the sucking, Moro and Babinski reflexes are frequently documented in the literature due to their important roles. The sucking reflex plays a critical role in oral feeding in coordination with breathing and swallowing, and is essential for nutrition intake for survival and growth. The Moro reflex is an involuntary protective motor response against abrupt disruption of body balance or extremely sudden stimulations [2]. The Babinski reflex involves the extensor and flexor of the foot as a nociceptive motor response of pyramidal tract [4]. Understanding these three reflexes might be more valuable in the neuro-behavioral examination of newborns considering their value for survival, protection, and development.

Therefore, the present study was undertaken to evaluate three representative primitive reflexes, the sucking, Moro and Babinski reflexes in Korean high-risk and preterm newborns. The specific study purposes were to describe various levels of their responses and to explore relationships among the primitive reflexes and various clinical conditions in high-risk newborns.

## Methods

### Study design and participants

This is a cross sectional explorative study with high-risk newborns recruited from September 2008 to June 2009 at the NICU of a university hospital located in Korea. Since primitive reflexes are usually present beginning at and after least 25 weeks of gestational age (GA) [2], newborns who were born at 25 weeks or older of GA were included for the study. Newborns were excluded if they had been transferred from other hospitals, to exclude possible missing information that was important to understand the clinical course. Newborns were also excluded if they had congenital, genetic or skeletal disorders; had received sedatives or the use of restraints; or had a maternal history of substance abuse or alcoholism, since these histories might influence the motor or neurologic responses.

### Data collection

Before data collection, the director of the NICU approved the study with the exemption of the informed consent since neurologic assessments particularly primitive reflexes, mental status assessment and behavioral status are part of the standard nursing procedures in the NICU. As well demographic and clinical information were anonymously obtained from existing medical records involving less than minimal risk without any additional data collection procedure. The researchers followed principles in the Declaration of Helsinki during the whole process of this study. The assessment of the sucking, Moro and Babinski reflexes were performed 48 hours after birth, to permit the newborns to become stable in the extrauterine environment after the usually necessary critical care had been provided. Newborns were examined while they were awake and lying comfortably on their backs. Examinations were avoided within 1 hour of feeding or direct contact with NICU staff or parents. Behavioral and mental status was assessed as a baseline state of newborns immediately before examining primitive reflexes.

### Measurements

The study variables consisted of clinical information, primitive reflexes, behavioral and mental status. Clinical information included demographics, birth history, and medical conditions such as respiratory condition, length of hospitalization (LOH), type of risk and brain sonogram. Respiratory status was categorized as completely self (independent), self with assistance such as oxygen therapy and continuous positive airway pressure (CPAP), and ventilator dependent. LOH was determined following discharge through medical record review. The risk group was categorized into; physiological risk group for simple preterm newborns without any pathologic conditions and pathologic risk group if pathological conditions were evident in addition to prematurity. Brain sonogram results were categorized as grade 0-IV (grade 0: normal result; grade I: hemorrhage limited to the germinal matrix; grade II: intraventricular hemorrhage without ventricular dilatation; grade III: intraventricular hemorrhage with acute ventricular dilatation; and grade IV: intraventricular hemorrhage extending into adjacent brain parenchyma) [5].

Evaluation of primitive reflexes for this study was based on the primitive reflex profiles Capute et al published [3], but did not follow their 5 point scale of 0 for absence to 4+ for pathologic prolonged response. Since Capute et al published the primitive reflex profiles in 1970s, high risk newborns of those days are quite different from those of these days. In addition, no further study was identified on the discriminative accuracy of 5-rating response. Therefore, we modified the 5 point scale to the 3 point scale indicating 0 for absence, 1 for abnormal and 2 for normal. Each reflex was examined up to five times if the newborn showed no response or if response was ambiguous to obtain best positive responses.

The newborns’ behavioral status was assessed using the Anderson Behavioral State Scale (ABSS) by observing five areas including the extent of eye openness, patterns of respiration, body movement, muscle tension and crying [6], and has been applied to high-risk newborns previously [7]. The score ranges from 1 to 12. Higher scores indicate a more alert state and 6 is a cut-off for an optimal alert state in infants [6]. The Cronbach’s alpha was 0.97 with Korean high-risk newborns previously [7]. The newborns’ mental status was evaluated using the Infant Coma Scale (ICS), which was developed by modification of the pediatric Glasgow Coma Scale (GCS), is a reliable tool to evaluate the mental status of high-risk newborns [8]. ICS evaluates the mental status of high-risk newborns in eye openness, verbal response and motor response. The total score of ICS can range from 3-15, and is interpreted just like GCS (i.e., higher score corresponding to a more alert state). Reliability and validity of ICS with Korean newborns displays a Cronbach’s alpha of 0.78 [8]. The performance and coding rule for each reflex, as well as the ABSS and ICS protocols, were confirmed before and during data collection to assure the validity and the reliability of the measurements.

### Data Analyses

The Statistical Packages for the Social Sciences version 18.0 was used for data analysis. Descriptive statistics and variable distributions were evaluated for data quality assessment. To describe clinical information and primitive reflexes, frequencies with percentages and means with standard deviations (SD) and ranges are presented. To determine relationships between primitive reflexes and other research variables, χ^2^, analysis of variance (ANOVA) and Bonferroni post hoc analysis was used with α = 0.05 in a two-tailed test.

## Results

A total of 63 newborns were included in the data analysis. Thirty three newborns (52.4%) were male and 11 (17.5%) were full-term newborns. Their mean GA of 33.6 (± 3.4) weeks (range: 25.9-40.7 weeks) and a mean birth weight (BW) of 2041.7 (± 706.3) grams (range: 806-3910 grams). At the time of assessment, the mean current age and current weight was 2.9 (± 1.5) days (range: 1-7 days) and 1983.8 (± 750.3) grams (range: 639-3900 grams), respectively. The sucking reflex presented a normal response most frequently (63.5%), followed by Babinski reflex (58.7%) and Moro reflex (42.9%). While one-third of newborns presented an abnormal Moro reflex (38.1%) or abnormal Babinski reflex (33.3%), only 11% of newborns presented an abnormal response of the sucking reflex. Absence responses were most frequent at the sucking reflex (25.4%), followed by the Moro reflex (19.0%) and Babinski reflex (7.9%). Most of the newborns were preterm births and displayed fair Apgar scores, good respiration, and minimal brain injuries in sonogram. The majority of the newborns had pathologic conditions, stayed long in the hospital, low ABSS scores and moderate ICS scores (Table 1).

**Table 1:** Clinical Information of the 63 Newborns

****	**Frequency (%)**	**Mean (SD)**	**Range**
Apgar score			
at 1 min		5.8 (2.1)	1-9
at 5 min		7.6 (1.8)	1-10
Length of hospitalization (day)		27.5 (24.5)	5-124
Brain sonogram*			
Normal	32 (50.0)		
Grade 1	21 (32.8)		
Grade 2	2 (3.1)		
Grade 3	2 (3.1)		
Grade 4	3 (4.7)		
Respiration			
Self only	45 (71.4)		
Self with assist	10 (15.9)		
Ventilator	8 (12.7)		
Preterm birth			
Yes	50 (74.6)		
No	13 (25.4)		
Risk group			
Physiologic	22 (34.9)		
Pathologic**	41 (65.1)		
ABSS		3.6 (3.4)	1-12
ICS		9.7 (1.9)	4-13

*The data of three newborns were missing; **the examples of pathologic risk group include respiratory distress syndrome, transient tachypnea of the newborn or intrauterine growth retardation.

We also explored relationships among primitive reflexes and clinical variables using χ^2^ analyses for categorical variables and ANOVA analyses for continuous variables. Babinski reflex showed different responses by brain sonogram results (χ^2^ = 16.56, P = 0.035). The sucking reflex (χ^2^ = 26.96, P < 0.001) and Babinski reflex (χ^2^ = 16.23, P = 0.003) were associated with respiratory condition, while the Moro reflex was associated only with the risk type (χ^2^ = 8.91, P = 0.012). Table 2 presented ANOVA analyses among the reflexes and continuous variables. While the sucking and Babinski reflexes concurrently presented significant associations with seven out of nine variables, the Moro reflex showed no relation with any of variables except ICS score (F = 8.37, P = 0.001). Newborns presenting a normal response of the sucking and Babinski reflexes were more likely to have older GA, heavier birth and current weight, shorter LOH, and higher Apgar scores and ICS scores. Current age and ABSS did not display significant patterns along with any response of the three primitive reflexes.

**Table 2:** Mean Difference of Continuous Clinical Variables by Primitive Reflex Reponses With ANOVA Analysis

****	**Sucking reflex**	**Moro reflex**	**Babinski reflex**
GA			
Normal^a^	34.6 (3.0)	33.9 (3.3)	34.5 (2.9)
Abnormal^b^	33.0 (3.4)	33.3 (3.7)	32.8 (3.8)
Absent^c^	31.2 (3.9)	33.7 (3.0)	30.7 (2.7)
F (P)	6.60 (0.002)	0.20 (0.822)	4.14 (0.021)
Bonferroni	a > c	—	a > c
Birth weight			
Normal^a^	2291.8 (647.8)	2065.5 (734.2)	2304.1 (686.2)
Abnormal^b^	1818.6 (667.2)	2022.3 (744.1)	1720.8 (569.1)
Absent^c^	1514.1 (553.2)	2026.7 (614.6)	1447.8 (507.1)
F (P)	9.27 (< 0.001)	0.03 (0.974)	7.94 (0.001)
Bonferroni	a > c	—	a > b, c
Current weight			
Normal^a^	2191.3 (674.5)	2012.3 (750.6)	2189.3 (721.2)
Abnormal^b^	1799.7 (694.8)	1988.6 (750.2)	1704.9 (609.8)
Absent^c^	1477.9 (569.3)	1809.6 (517.2)	1614.7 (946.8)
F (P)	6.33 (0.003)	0.28 (0.760)	3.80 (0.028)
Bonferroni	a > c	—	—
LOH			
Normal^a^	19.9 (19.4)	22.6 (17.0)	19.6 (15.3)
Abnormal^b^	28.6 (25.5)	32.6 (31.4)	40.6 (32.5)
Absent^c^	49.6 (25.4)	35.0 (25.8)	47.0 (23.2)
F (P)	10.69 (< 0.001)	1.44 (0.245)	7.19 (0.002)
Bonferroni	a < c	—	a < b
Apgar score at 1 minute			
Normal^a^	6.3 (2.0)	5.9 (2.2)	6.3 (2.1)
Abnormal^b^	6.6 (1.3)	5.8 (2.1)	5.6 (1.6)
Absent^c^	4.4 (2.0)	5.7 (1.8)	3.4 (2.4)
F (P)	5.59 (0.006)	0.05 (0.955)	5.13 (0.009)
Bonferroni	a, b > c	—	a > c
Apgar score at 5 minute			
Normal^a^	8.0 (1.6)	7.7 (2.1)	8.1 (1.5)
Abnormal^b^	7.9 (0.7)	7.4 (1.8)	7.3 (1.4)
Absent^c^	6.4 (2.3)	7.8 (1.1)	5.2 (3.6)
F (P)	4.78 (0.012)	0.17 (0.842)	6.72 (0.002)
Bonferroni	a > c	—	a > c
ABSS			
Normal^a^	3.9 (3.4)	4.0 (3.6)	4.2 (3.6)
Abnormal^b^	5.1 (4.1)	3.2 (2.9)	2.9 (2.9)
Absent^c^	2.4 (2.7)	3.8 (3.9)	3.0 (3.4)
F (P)	1.99 (0.146)	0.37 (0.690)	1.11 (0.337)
ICS			
Normal^a^	10.3 (1.3)	10.2 (1.5)	10.3 (1.4)
Abnormal^b^	8.6 (3.6)	9.9 (1.5)	8.5 (2.6)
Absent^c^	8.4 (2.1)	7.7 (2.9)	9.4 (.5)
F (P)	7.11 (0.002)	8.37 (0.001)	6.03 (0.004)
Bonferroni	a > c	a, b > c	a > b

GA: Gestational age; ABSS: the Anderson Behavioral State Scale; ICS: the Infant Coma Scale; LOH: length of hospitalization; ANOVA analysis between current age and reflexes was removed due to no association.

## Discussion

Assessing primitive reflexes is an important part of high risk newborns. Primitive reflex responses in premature newborns may often vary in degree of responses. However clinicians have mostly using dichotomous criteria of presence or absence of primitive reflexes without guide or research based evidence in high-risks. It is also unclear how the degree of their response should be described in detail and how much the information could be meaningful clinically. This study was conducted to describe primitive reflexes assessed in 63 Korean high-risk newborns. After review of details in each primitive reflex, three particularly noteworthy findings warrant further comment.

First, a considerably high number of high-risk newborns (36%) presented an abnormal or absent sucking reflex. The sucking is a survival reflex that is observed as early as GA 25-26 week and typically before 28 weeks, although intrauterine swallowing activity begins as early as 12 week of GA [1]. Absence of the sucking reflex in 25.4% of the newborns was an unexpectedly substantial finding considering that their mean GA was 34.6 weeks. A possible explanation is that their clinical condition, such as difficult respiration and decreased mental status, may have delayed sucking reflex response. Premature newborns who are ventilated can exhibit significantly poor sucking ability [9]. The presence of nasal CPAP or endotracheal tube, as well as oro-gastric tube if indicated, could alter perioral activities [10]. Therefore, the newborns in this study may have developed a similar alteration or habituation from strenuous nasal or perioral stimulation, which was responsible for the absence of the sucking response. Furthermore, although the associations between brain sonogram findings and reflexes were not identified, the mental status as assessed by ICS showed a statistically significant association with the sucking reflex. Particularly, the mean ICS score of the newborns presenting an abnormal or absent sucking reflex response < 9, which is a cut-off point for the normal mental status of infants [8]. In a previous study, sucking behavior was shown to provide an indirect indicator of maturity of neurological development in premature newborns [11]. Therefore, an abnormal or absent sucking reflex may imply a neurologic impairment in the high risk newborns in this study. This statement is consistent with their longer LOH.

Secondly, among the three primitive reflexes, the Moro reflex presented quite different patterns with the clinical conditions of the newborns from the other two reflexes, while very similar response patterns were observed in the sucking and Babinski reflexes. Moro reflex presented as the least frequent normal response and most frequent abnormal response. These observations are consistent with previous studies indicating that the Moro reflex is especially weak in preterm newborns because of lower muscle tone, poor resistance to passive movements and slow arm recoil, compared with those of full term newborns at same post-conceptual age [12]. In our study, morbidity-related factors such as LOH, Apgar scores, and ICS total scores were statistically associated with the sucking and Babinski reflexes, but not the Moro reflex. This data may refer that the Moro reflex is more likely related to infant development rather than pathologic conditions.

Lastly, through this study we tried to highlight the main issues of whether the presentation of an abnormal response(s) of the primitive reflexes in the high-risk newborns is bad, and, which of the reflexes carries a greater clinical importance in the case of an abnormal or absence response. In this study, there were significance differences of clinical conditions between normal and the other responses. Although most of the posthoc analyses have not presented any differences of clinical condition between abnormal and absent responses, abnormal response seems as much adverse as absence response. This phenomenon is more obvious in the sucking and Babinski reflex. Therefore, it might be more prudent to assess them separately and search for an explanation through further research.

How do we apply this evidence to our practice? Assessing primitive reflexes is a part of standard care in NICUs which is easy to evaluate and feasible to perform in any circumstances without high technology equipment. Although many of clinicians understand the importance and advantages of primitive reflex assessment and have assessed them to determine if they are present or absent, clinicians often disregard abnormal responses which high-risk newborns often present. Subtle changes of primitive reflex could be valuable indicators of current medical conditions and potential future health and developmental outcomes while newborns grow up. It would be beneficial to develop standardized assessment protocol for primitive reflexes in high-risk infants using at least four scales (absence, hypoactive, normal, and hyperactive) as assessment of adults’ reflexes. Further study is also recommended in this understudied area. It would be more beneficial to assess variety of primitive reflexes using more detail assessment criteria. For example, the sucking reflex may be differently categorized by its presence, frequency, regularity, pressure and coordination with breathing or swallowing. Some reflexes, such as the Babinski reflex, might present different responses bilaterally. In that case, assessing whether responses are consistent bilaterally may reflect more differentiated aspects of clinical conditions.
